# Developing quality indicators for in-patient post-acute care

**DOI:** 10.1186/s12877-018-0842-z

**Published:** 2018-07-11

**Authors:** John N. Morris, Katherine Berg, Eva Topinkova, Leonard C. Gray, Erez Schachter

**Affiliations:** 1 Institute for Aging Research, Hebrew Senior Life, Boston, USA; 2 University of Toronto, Toronto, Canada; 3 Physical Therapy Centre of Excellence in Health Services/Health Policy Research and Training (CoHSTAR), Brown University, Providence, USA; 4 Department of Geriatric Medicine, First Faculty of Medicine, Charles University, Prague, Czech Republic; 5 Faculty of Health and Social Sciences, South Bohemian University, Ceske Budejovice, Czech Republic; 6 Geriatric Medicine at the University of Queensland Centre for Research in Geriatric Medicine, Brisbane, Australia; 7 Profility Inc., Boston, USA

**Keywords:** Post-acute care, PAC, Skilled nursing facility, SNF, Quality Indicator, Post-acute quality indicator, Quality indicator standard, Post-acute quality indicator summary scale

## Abstract

**Background:**

This paper describes an integrated series of functional, clinical, and discharge post-acute care (PAC) quality indicators (QIs) and an examination of the distribution of the QIs in skilled nursing facilities (SNF) across the US. The indicators use items available in interRAI based assessments including the MDS 3.0 and are designed for use in in-patient post-acute environments that use the assessments.

**Methods:**

Data Source: MDS 3.0 computerized assessments mandated for all patients admitted to US skilled nursing facilities (SNF) in 2012. In total, 2,380,213 patients were admitted to SNFs for post-acute care. Definition of the QI numerator, denominator and covariate structures were based on MDS assessment items. A regression strategy modeling the “discharge to the community” PAC QI as the dependent variable was used to identify how to bring together a subset of seven candidate PAC QIs for inclusion in a summary scale. Finally, the distributional property of the summary scale (the PAC QI Summary Scale) across all facilities was explored.

**Results:**

The risk-adjusted PAC QIs include indicators of improved status, including measures of early, middle, and late-loss functional performance, as well as measures of walking and changed clinical status and an overall summary functional scale. Many but not all patients demonstrated improvement from baseline to follow-up. However, there was substantial inter-state variation in the summary QI scores across the SNFs.

**Conclusions:**

The set of PAC QIs consist of five functional, two discharge and eight clinical measures, and one summary scale. All QIs can be derived from multiple interRAI assessment tools, including the MDS 2.0, interRAI-LTCF, MDS 3.0, and the interRAI-PAC-Rehab. These measures are appropriate for wide distribution in and out of the United States, allowing comparison and discussion of practices associated with better outcomes.

**Electronic supplementary material:**

The online version of this article (10.1186/s12877-018-0842-z) contains supplementary material, which is available to authorized users.

## Background

Quality indicators have tended to be country specific in origin. In the United States, for example, the Centers for Medicare and Medicaid (CMS) report a diverse set of quality measures for long stay residents and a more limited set for short stay patients on Nursing Home Compare [1]. The Improving Medicare Post-acute Transformation Act required skilled nursing facilities (SNFs) to report 30-day re-admission rates and successful discharge to the community [2]. Currently the only CMS improvement measure for short stay patients in SNF focuses on change from admission to discharge in walking, locomotion or transfers – mid-loss Activities of Daily Living (ADLs). Yet, almost all patients who enter an in-patient PAC will have recently experienced a more comprehensive functional loss [3, 4]. Basic activities such as dressing, toileting, and even walking will now require the help of others – at least during their period of recovery in post-acute care. Many patients will also have a complex set of clinical complications, including delirium, cognitive and communication decline, pain, pressure ulcers, and mood distress [5, 6]. Nevertheless, their placement in an in-patient post-acute care setting represents a sign of hope. The losses these people have experienced are almost always of more recent origin and this placement decision suggests that the acute hospital team expect to see stabilization and even improvement in patient status. And to that end, Skilled Nursing Homes (or SNFs) are a common post-acute option in the US, and can be expected to provide an aggressive program of recuperative and rehabilitative services.

A broader array of quality indicators would be beneficial in assisting facilities to improve care in multiple areas. Moreover, quality indicators based on admission to first assessment are valuable because the time frame is the same for all patients and they reflect early changes or lack thereof that may influence longer term outcomes such as successful discharge. The early QIs would encourage facilities to monitor early outcomes – e.g., those within two weeks of admission and thereby improve longer term outcomes.

Following the work for CMS that informed the Nursing Home Compare quality indicators [7], Morris and associates have continued to build an array of quality indicators for long stay [8] which have in turn informed the quality indicators used in Canada by Canadian Institute for Health Information (CIHI) and elsewhere (e.g., Finland, Belgium, and New Zealand). They have subsequently addressed the need to develop post-acute quality indicators [9].

This paper extends this prior work by describing the development and refinement of a comprehensive set of individual and summary quality indicators to be used to track the recovery trajectory of patients served in in-patient post-acute care (PAC) settings, particularly those which use interRAI based assessments such as the MDS 2.0, MDS LTCF, and MDS 3.0. True global standards do not currently exist and in this paper, using the largest post-acute data set ever assembled, we innumerate a set of post-acute care quality indicators (PAC QIs) and examine their distribution across SNFs in the US.

PAC patients will have begun their recovery in the acute hospital, many can be expected to continue to improve over the typical four week PAC in-patient stay [4, 6, 10]. The PAC in-patient care setting, like the acute hospital before it, is not intended to be a care environment in which full functional and clinical recovery can be expected. Rather the PAC is best described as a “way station” where incremental improvement is possible. The profile of pervasive functional disabilities at admission is translated into one of a more engaged patient at the time of discharge.

In this light the PAC QIs described in this paper provide a set of measures against which to monitor the relative success or failure of in-patient PAC care settings in expediting partial to even full patient recovery. Our outcomes track functional improvement, clinical recovery, return to the community, and for those who did not improve discharge back to a hospital.

CMS has identified issues of global recovery across the whole stay including successful discharge to the community [2]. Others have articulated a number of more problem-specific outcome measures such as stroke, joint replacement, falls, pulmonary care, wound care, coronary bypass follow-up, and hip fracture [5, 11–18]. In our review of this work the major outcomes of interest have included functional improvement, length of stay, discharge status, re-hospitalization, and cost of care. Other measures include return to the person’s pre-episode functional condition, survival, delirium, falls, pressure ulcers, and pain. Most of these measures have been displayed as single indicators that cover the full post-acute stay – entry to discharge measures. Few have put forth measures that relate specifically to the early and mid-parts of the stay. For example, functional measures typically focus on changes that occurred from admission to discharge, with efficiency scores calculated based on the change in the functional measure divided by the length of stay [19]. And while CMS has identified a number of quality measures for public reporting in rehabilitation hospitals (including influenza vaccinations, new and worsening pressure ulcers, catheter-associated urinary tract infections, and re-hospitalizations within 30 days of discharge from post-acute care [2]), there are no requirements to document changes at fixed points during the course of the stay, only at admission and discharge. Thus, our establishment of PAC QI standards at 14-days and 30-days into the stay opens up a new and important area of inquiry. Now, early into the stay one can assess how well PAC in-patient care sites are responding to the needs of the patients.

## Methods

### Sources of data/study population

The data set used in this paper consisted of all SNF MDS 3.0 assessments for Medicare patients admitted during calendar year 2012 in the US. Included are 2,380,213 admission, of those 1,852,913 have a 14 day follow up and 1,852,218 have a second 30-day follow-up assessment. These occurred in 15,042 SNF sites – translating into an average of 158 admission assessments per SNF. Additional inclusion criteria were a second MDS 3.0 assessment within 30 days of admission or a rehospitalization from SNF during the SNF stay.

Pursuant to CMS rules that have been in place for over 25 years, trained clinical staff – almost always a nurse or an assessment team lead by a nurse complete the MDS 3.0 assessments. The MDS 3.0 includes a diverse array of descriptive and judgmental items – including functional status, cognitive status, likelihood for functional improvement, mental health status, disease diagnoses, clinical status, and a judgement of likelihood to return to the facility following discharge – to name a few. Physical function includes patients’ performance of basic the Activities of Daily Living (ADL): personal hygiene, dressing, locomotion, transfer, toileting, bed mobility and eating. Each of the ADLs is assessed across a five-point dependency scale. The first two categories (scored as “0” and “1”) referenced patients who were independent or required only supervision (but no physical support); while the three latter categories (scored as “2,” “3,” and “4”) referenced patients who received physical assistance of varying degrees from others. CMS provides a detailed instructional manual for completing the items and following the required schedule of assessments: 5 day, 14 day, 30 day, 60 day, 90 day or discharge. The MDS assessments completed by the facility assessors have been shown to be reliable, accurate, and valid [20–23]. The primary intended use of these assessments is to guide care planning and to monitor changes in status. But the MDS 3.0 assessments also form the basis for prospective payment in SNFs and thus, the quality of the data is closely monitored by CMS.

These items also occur in other interRAI assessment instruments (the MDS 2.0, the interRAI-LTCF, and the interRAI PAC Rehab). Embedded in the assessments are several scales including the Cognitive Performance Scale and the ADL Long Form [20, 24].

#### Profiles of patients admitted to SNFs

We examined the distribution of ADL item performance at admission based on the interRAI-developed functional items found in the MDS 3.0. We next used these same ADLs to assess how PAC patients changed by the time of the first follow-up at or about day 14 into the stay. In the MDS 3.0 the US included a specific requirement that even discharged patients had the follow-up items (functional as well as clinical) assessed. We also looked at the proportion of patients who had returned to the community.

Finally, based on the prevalence of the candidate QI clinical complication measures at the baseline and first follow-up assessments we determined which conditions had a reasonable prevalence and how were they likely to change during the course of the PAC stay.

### Development and refinement of PAC QIs

Using MDS 3.0 data, each of the facility PAC QI measures were based on the total cohort of patients served over a 12-month period. For the PAC environment we had previously used a three month window [9], but with the existence of many small SNF programs the three-month window resulted in large numbers of SNFs being unable to meet the minimal sample size requirement for the denominator of each QI – 20 cases. Functional and clinical PAC QIs were based on all patients with an MDS assessment at about days 14 and 30 respectively. For the discharge QIs we drew on all patients who had been discharged either through day 14 or cumulatively through day 30.

For a facility PAC QI estimate to be calculated, 20 or more patient assessments had to be available at the follow-up assessment. Let us say, for example, that 50 PAC patients were admitted to a site during the year, then for us to display the 14-day functional and clinical PAC QIs at least 20 of these patients would have to have a 14-day assessment. For the discharge QIs, on the other hand, all we would have to know was the discharge status (discharged or not) of the patients (50 in this example) at the time of the two follow-ups.

Using this standard, and depending on the specific PAC QI measure, outcome estimates were created for between 12,342 SNFs and 14,193 SNFs – or 82 to 94% of all SNF sites in the US in 2012. The remaining SNF sites, where individual PAC QIs were not available, did not have a sufficient number of patients at the designated follow-up assessment (14 days or 30 days) on which to base a reasonable estimate for a specific quality indicator.

For the functional PAC QIs, we stayed close to our prior constructs, following the general model laid out by Sidney Katz [25] for describing the stages of functional loss. Our measures reference three tiers of ADL loss that set the scope of our improvement PAC QIs. The first tier references dressing and personal hygiene, followed by locomotion and transfer, and ending in independence in toileting, moving in bed, and feeding oneself.

In a PAC setting, choices are made with respect to physical and occupational therapy targets. Thus, we would expect to observe inter-facility variation in the improvement profiles for these three types of ADL targets. Said another way, looking through the lens of how staff perceive the needs of the person and the expectation of return to the community (where it is likely that rehabilitation therapy will be continued), program staff may place more or less emphasis on improvement in areas seen to expedite patient goals and ultimate return to the community. Some may focus on walking while others may focus on personal hygiene.

Sites may or may not do equally well in addressing the functional losses in these areas, and by having multiple ADL-QI measures, this performance variation can be tracked. From this viewpoint, we created quality indicators addressing each of the three ADL tiers: our variation of an early loss measure based on personal hygiene and dressing, mid-loss based on locomotion, transfer and toileting, and late loss based on bed mobility and eating. Any improvement would be considered the outcome. In addition, we created two other ADL PAC QIs. First a single measure of improvement in walking (note - the mid-loss ADL references locomotion by any means item versus the moving only by walking item). Finally, we included a single summary functional PAC QI, a measure that is based on the ADL Long Form Scale [20]. Of all of our ADL measures, this was the only one in which we required a two or more point improvement to indicate that a meaningful change had occurred -- in our view lending credence to the belief that the patient had begun to assume more responsibility for his/her own activities of daily living. For the three ADL stage quality measures and the walking measure, we assessed whether the person exhibited any improvement (i.e., a one or more point change).

The selected clinical PAC QIs reference common conditions that have been suggested to be relevant to patients who have experienced significant functional loss. These clinical PAC QI measures include: pain; mood; pressure ulcer; unsteady gait; shortness of breath; and delirium. The two remaining clinical PAC QIs reference not improvement in status, but the presence of a problem at follow-up. They are falls and indwelling urinary catheter use.

The two final individual PAC QI measures reference patient discharge status: discharge to the community or discharge to a hospital.

Table 1 displays the areas covered by the PAC QIs. The first column indicates the name of the PAC QI. The second column further clarifies the QI definition. For all of these QIs we created two measures, one at the first follow-up (scheduled at day 14) and one at the second follow-up (scheduled at day 30).

**Table 1:** PAC Individual Quality Indicators

PAC-Individual quality indicators	Nature of measure at 1st and 2nd follow-ups
Early-Loss ADL	% Who Improve or Remain Independent
Mid-Loss ADL	% Who Improve or Remain Independent
Late-Loss ADL	% Who Improve or Remain Independent
ADL Long Form	% Who Improve by 2 or more points
Walking	% Who Improve or Remain Independent
Indwelling Catheter Use	% Who Use
Falls	% Who Fall
Pain	% Who Improve or Are Pain Free at Follow-up
Mood	% Who Improve or Are Free of Depression at Follow-up
Pressure Ulcer	% Who Improve or Who Are Free of PU at Follow-up
Unsteady Gait	% Who Improve or Are Problem Free at Follow-up
Shortness of Breath	% Who Improve or Are Problem Free at Follow-up
Delirium	Problem Free at Follow-up
Discharged to Community	Percent Discharged to Community
Discharge to Hospital	Percent Discharged to Acute Hospital

### The covariate adjustment process

It is unreasonable to expect all SNFs to admit the same profile of patients. There will be differences in age, functional status, cognition, and clinical case-mix. Thus, if we were to limit our QIs to raw change measures, we fear that those who might wish to use our PAC QIs would be rightfully concerned with the QI forms. In such a situation the differences in the observed PAC QIs could be due solely to variations in the admission case-mix across the SNF facilities. For example, persons with significant cognitive loss are likely to have slower rates of recovery than are persons who have no cognitive deficits, and a site with many more such patients may be expected to have lower rates of recovery [26].

To counter this concern, we have introduced covariate adjustment processes [9, 27]. With this type of strategy “adjusted” PAC QI estimates are created, resulting in a measure that has been adjusted up or down based on the covariate distribution of the statistically identified relevant patient variables that are related to variation in the QI measure. As a simple example, if cognition was negatively correlated with ADL recovery and a SNF site had many more cognitively impaired patients than the typical site then that SNF site should on average do poorer than other sites. Thus in the covariate adjustment process the observed ADL improvement QIs for sites with more cognitively impaired patients would receive a slight boost in their score – an adjustment toward better ADL QI scores commensurate with the sites’ average cognitive score vs. the average cognitive score of all of the sites. For the adjustment to occur there has to be a correlation between the adjustor and the raw PAC QI and the adjustor has to have a distribution that differs across SNF sites.

By adopting this covariate adjustment approach we can say that the PAC QIs being used are displayed across a relatively “level playing field.”

In our current work, with an enormous national sample, we were able to create a rather comprehensive array of covariates. Among the measures considered were the full complement of individual ADLs (including a number of dichotomous forms for each measure), use of appliances, the Cognitive Performance Scale (with a number of dichotomous forms for this measure as well), a clinical severity scale, a wide variety of diagnoses (including ALS, MS, hemiplegia, paraplegia, quadriplegia, stroke, CHF), bladder continence, bowel continence, and behavior problems.

The covariate measure pool included patient characteristics that could differ across sites. All of the items in this pool were derived from the extensive item set found in the US MDS 3.0 assessment instrument. At the same time as we considered these items as possible covariates we made the decision not to consider measures that reflected service use at the SNF sites. For example, we excluded items that reflected the rehabilitation service process. Such items reflect the SNF process of care and one would not wish to exclude such variation from the PAC QI measures.

The logistic multivariate regression covariate modeling process occurred separately for each PAC QI with discrete models for the 14-day and 30-day models. Through this process we selected covariates that had a reasonable clinical relation to the dichotomous PAC QI scores (e.g., the proportion of persons who improve in Early-Loss ADL). At the same time, with an enormous national cohort, we ran the risk of entering covariates that had very small odds ratios. Such covariates would have no meaningful adjustment effect on the quality indicators. To avoid this from occurring, we set what appeared to us to be reasonable a priori minimum odds ratio values for a measure to enter the equation: 1.2 or higher or 0.799 or lower. There is no one agreed upon standard for inclusion of items but these values have the advantage of suggesting that sites may actually differ in some measurable way in the confounding measures. [Note, although these models are not reported in detail in this paper, they are available from the lead author.]

### Creation of summary QI measure

Next we created a Summary PAC QI measure, providing a single global overview of each SNF site’s quality performance. The work in creating this measure involved a three-stage process. First, each PAC QI was converted into a standardized z-score measure (where each raw score is subtracted from the variable mean and divided by the variable standard deviation) and arrayed so that the high end represented the positive outcome. Then each of these score ranges was divided into three equal sized categories, scored from “0” to “2,” using the measures standard deviation to create the roughly equaled sized groups. Next, we correlated each measure against the discharge to the community (home) PAC QI.

Measures with a “positive” association with the discharge QI went forward into the next step. Here we divided the sample into two random halves and completed two separate multiple logistic regressions to identify the final set of variables to enter the summary QI: one to make preliminary decision on covariates and the other to validate the decisions, based on the consistency of results. Only the measures that were present in the two regressions entered the final global quality measure.

Through this process our goal was to create one PAC QI summary scale, and this approach permitted us to select the maximum set of the individual PAC QIs that held together. The appropriateness of this final summary scale was tested with the KR 20 Alpha reliability statistics – a measure that indicated the extent to which there was a consistent pattern of positive correlations among the items in the scale.

Finally, the variation of the average SNF facility score on the summary PAC quality scale was reviewed by state. The large sample of patients and facilities permitted an estimate of benchmark standards at the 20,50 and 80th percentiles for post acute facilities.

### Ethics

The data used were provided pursuant to an agreement with CMS in the United States. The analyses are covered by an approval from the Hebrew Senior Life, Institute for Aging Research, Institutional Review Board, and the analyses were completed using SPSS version 20 and 22.

## Results

As indicated earlier, post-acute in-patient care occurs largely in the month following admission to the SNF and this pattern is confirmed in these US data. In our cohort, by 30 days into the stay only 30.5% of the patients were still in the SNF, 41.2% had been discharged to the community, 22.2% had been discharged back to an acute hospital, 2.9% had died, 2.3% had entered a long term-care facility, and less than 1% had gone to another setting.

### Functional profile of patients admitted into SNFs

**Fig. 1:**
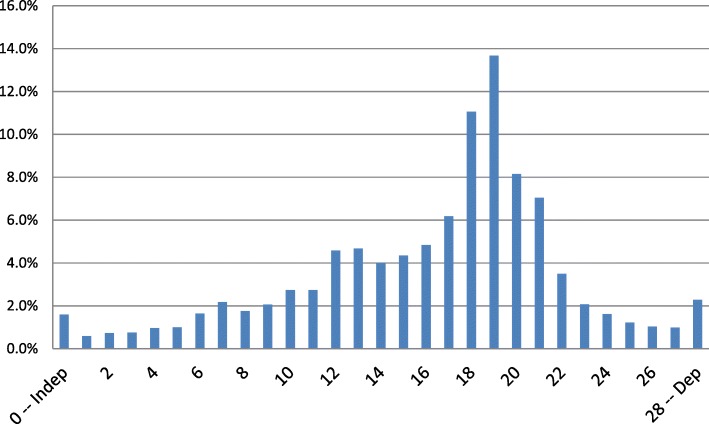
ADL Long Form Distribution at Time of the Baseline (5-Day) Assessment (Mean = 17.8)

Table 2 displays the individual ADL measures at the time of the SNF admission assessment – before significant rehabilitation services could have been provided, while Fig. 1 displays the distribution across the ADL Long Form Scale.

**Table 2:** Percent of Patients Scoring at each Level for ADL Tasks at the Day Five Assessment

ADL	Independent (0)	Supervision (1)	Limited Assistance (2) – patient highly involved	Extensive Assistance (3) – Staff provide weight bearing help	Total Dependence (4)
Personal Hygiene	4.1	7.6	22.0	56.9	9.4
Dressing	1.8	3.4	17.4	68.7	8.7
Transfer	2.1	3.9	17.3	65.5	11.2
Locomotion	5.2	7.7	19.3	44.1	23.7
Toilet Use	2.1	3.4	15.0	67.7	11.8
Bed Mobility	4.2	4.1	16.1	68.9	6.7
Eating	33.5	35.8	12.8	12.0	5.9

For the individual ADL measures, the pattern at this early point in the stay is generally one of rather extensive dependency on others. The modal patient response category, with the exception of eating, was extensive assistance – about two-thirds of the PAC patients were in this category. Only small numbers of patients were independent in one or more of the ADL areas. For example, 95% of patients required help from others in dressing and toilet use.

In Fig. 1, which displays the distribution of ADL Long Form Scale scores at admission, the mean score was 17.8 (on a scale that ranged from 0 to 28). Few patients were totally dependent or totally independent. Said another way, 96% of all SNF patients at the time of admission depended on others for physical help in completing their ADLs.

### Length of stay and changes in functional status

**Fig. 2:**
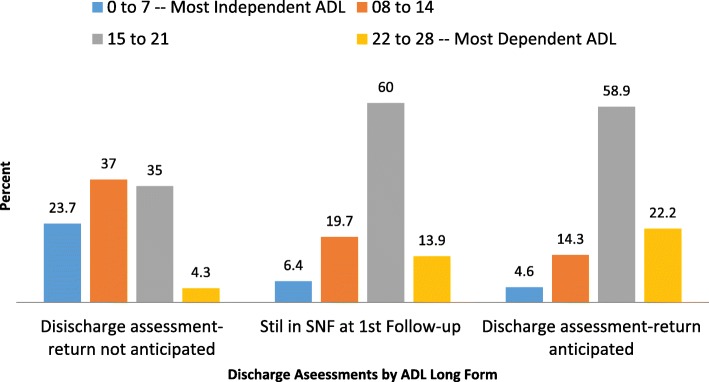
Discharge Status at 1st Follow-up By ADL Long Form Score at 1st Follow-up

By the time of the first follow-up, at about 14 days into the stay, patient status began to change. Figure 2 displays how the ADL Long Form Scale changed as patients moved from or remained within the SNFs between the baseline and first follow-up assessment.

The average change in the ADL Long Form score paralleled patient movement (or lack thereof) between the baseline and first follow-up assessment. For discharged patients where the MDS assessor said the person could be expected to return to a SNF, there was little change in the ADL Long Form Scale score. The ADL profile was about the same as those who were still in a SNF PAC. These patients who remained in the SNF, demonstrated one point change in the average Long Form Scale score – from a mean of 18.1 to a mean of 17.0.

For patients who were discharged to the community and who were judged by the MDS assessor to be unlikely to return to a SNF, the mean ADL Long Form Scale score improved – from an average score of 15.2 to an average score of 12.5. Their average score at admission was 2.6 points below the average of all PAC patients and by the first follow-up they had a further average improvement of 2.7 points.

Looking next at the eight clinical areas, there was a baseline average mean problem prevalence of 15%. Pain came in as the highest at 28%. From baseline to the 14-day assessment, a return to a problem free status was somewhat common for pain (9%), shortness of breath (5%), and falls (5%). New incident events were most common for delirium (6%), pain (4%), falls (4%), and shortness of breath (3%).

#### Facility level quality indicators and covariates

Table 3 lists the covariates for each PAC Qi that were identified through multivariate logistic regressions.

**Table 3:** PAC Individual Quality Indicators AND Their Covariates

PAC-Individual quality indicators	Covariates
Early-Loss ADL	Use of a mobility device, bladder continence, bowel continence, Cognitive Performance Scale (CPS), unsteady gait, walk in room, eating, toilet use
Mid-Loss ADL	CPS, unsteady gait, eating, personal hygiene
Late-Loss ADL	Mobility device use, bladder continence, CPS, unsteady gait, walk in room, personal hygiene,
ADL Long Form	Bladder continence, bowel continence, CPS, unsteady gait
Walking	Bladder continence, bowel continence, CPS, pressure ulcer, plegia (hemiplegia, paraplegia, tetraplegia)
Indwelling Catheter Use	Patient severity index, amyotrophic lateral sclerosis (ALS), CPS, pressure ulcer, transfer, toilet use, walk in room, personal hygiene, paralysis
Falls	CPS, behavior, transfer, hip fracture
Pain	CPS, bed mobility, hip fracture, stroke
Mood	Age, CPS, behavior
Pressure Ulcer	Congestive heart failure, transfer, walk in room, locomotion on unit, eating, paralysis, stroke
Unsteady Gait	Bowel continence, transfer, walk in room, dressing, eating, personal hygiene
Shortness of Breath	CPS, swallowing problem, transfer, locomotion on unit, hip fracture, stroke
Delirium	CPS, behavior, swallowing problem, eating, personal hygiene,
Discharged to Community	Mobility device use, bladder continence, congestive heart failure, CPS, unsteady gait, walk in room, personal hygiene, walk in room, stroke
Discharge to Hospital	Mobility device use, bladder continence, congestive heart failure, CPS, unsteady gait, personal hygiene, walk in room, stroke

The average PAC QI had 8 covariates, the range was from 5 to12 covariate adjustors. Twenty-three individual covariates appeared in one or more of the PAC QIs. Cognitive impairment, as represented by the Cognitive Performance Scale [28], was present as an adjustor in 77% of the QIs. Covariates appearing as adjustors in at least 30% of the PAC QIs included walking, the other individual ADL items, use of an assistive mobility device, bladder continence, bowel continence, a patient summary clinical severity measure, and gait problem. Covariates appearing in fewer PAC QIs included congestive heart failure, pressure ulcers, a behavior problem, hip fracture, stroke, amyotrophic lateral sclerosis (ALS) diagnosis, and age. (List of coefficients and weights are available from lead author on request).

For this set of PAC QIs, when assessed at the two follow-up points, the average correlation between the raw and adjusted PAC QI measures is 0.924 – the high was 0.99 and the low was 0.80. The net result is that the PAC QIs estimated for some SNF sites moved up slightly, while the PAC QIs for other sites moved down a little – on average we saw a movement of about 29% of one standard deviation across all the PAC QIs.

#### Distribution of PAC QI scales

**Fig. 3:**
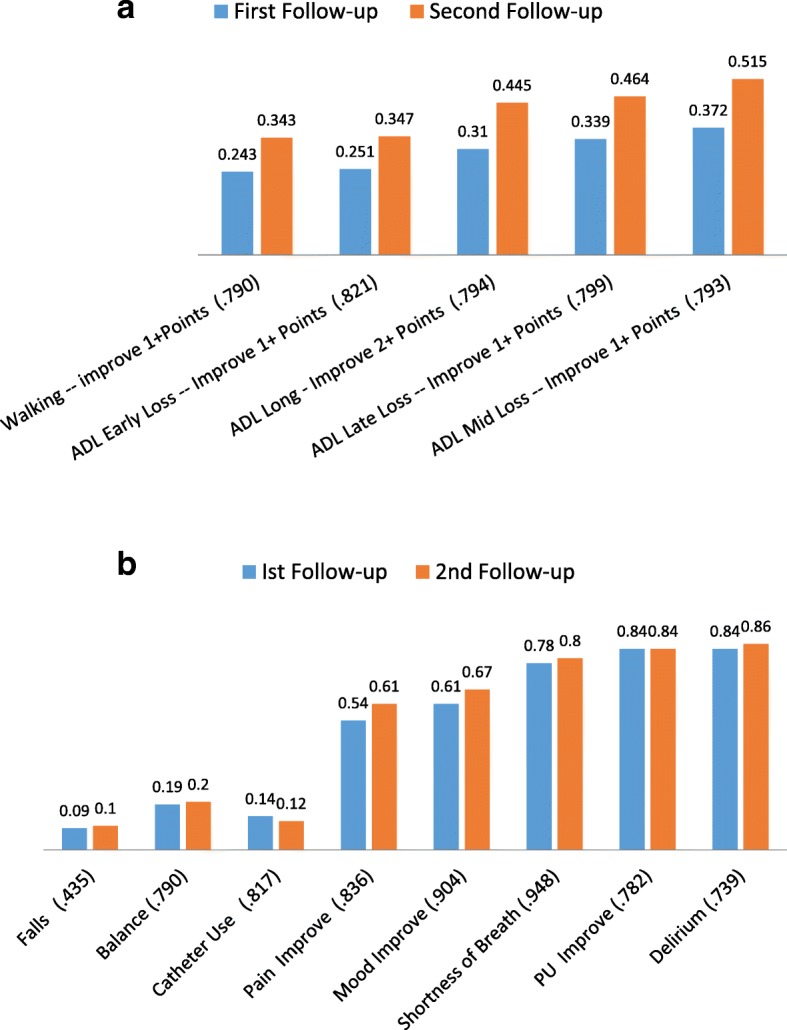
**a** Functional QIs – Adjusted Rates at 1st and 2nd Follow-ups (Correlation Between 1st and 2nd Follow-up QI) **b**. Clinical QIs – Adjusted Rates at 1st and 2nd Follow-ups (Correlation Between 1st and 2nd Follow-up QI)

Figure 3a and b present the covariate adjusted PAC QI rates for the US at the 14-day and 30-day follow-ups [note, the Additional files 1 and 2 includes information on the expected facility averages for each PAC QI at the 20th, 50th, and 80th percentiles – these in our view represent reasonable points to identify better and poorer performing SNF sites].

Figure 3 shows that the ADL Pac QI rates rose over time but were greatest for mid-loss – which includes locomotion and the transfer items. Thirty-seven percent of patients still in the facility at the first follow-up had improved, while the number rose to 52% by the second follow-up. For the ADL Long Form Scale where we assessed improvement by two or more points, 31% of patients improved by two or more points by the first follow-up, while 45% improved by the second follow-up (two weeks later).

In addition, the facility-level correlations between the 14 day and 30 day facility PAC QI estimates were quite high – with an average correlation of 0.80. This suggests that SNF facilities that tended to do well at 14 days also tended to do well at 30 days, while facilities that did poorly at 14 days also tended to do poorly at 30 days.

Of the eight clinical PAC QIs (Fig. 3b), only the rates for Pain and Mood improved over time. All other clinical PAC QI rates remained about the same at 14 and 30 days into the stay. Falls and Catheter use are negative prevalence PAC QIs – with rates of 9 and 14% respectively. All of the other clinical PAC QIs are defined to reflect improvement or remaining problem free. The Pain and Mood clinical QIs sit in the middle with about one-third who failed to improve by the second follow-up. Finally, shortness of breath, pressure ulcers, and delirium each had about 15 to 20% who did not improve by the second follow-up.

**Fig. 4:**
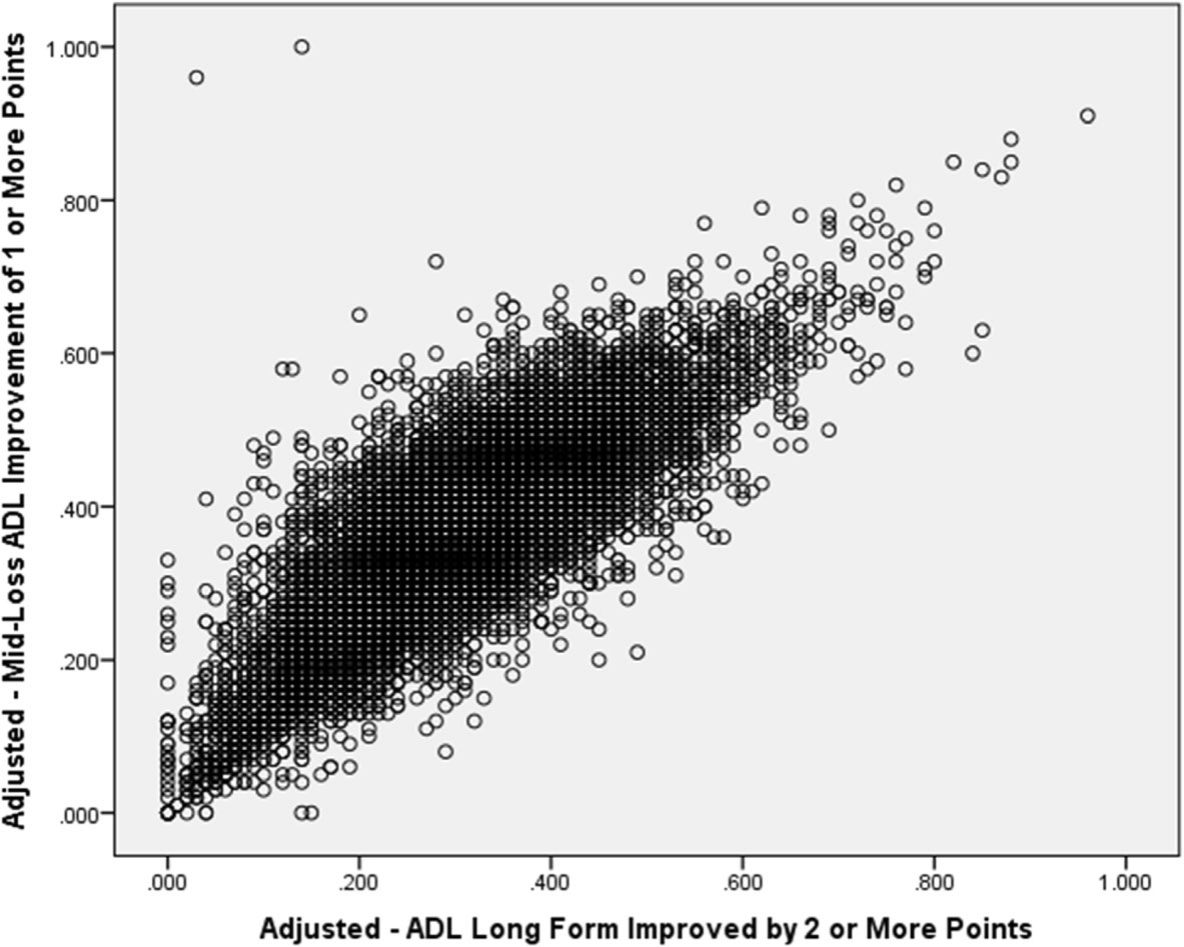
Correlation of SNF Adjusted QI Scores for Mid-Loss ADL and ADL Long Form Measures

Figure 4 displays the adjusted values for Mid-Loss ADL and ADL Long Form improvement PAC Qis. Although quite strongly related, the correlation coefficient of 0.82 is not as high as might be expected given that mid-loss ADL tasks are included in the total ADL Long Form QI, suggesting that facilities differ somewhat in how well they perform on the individual ADL Qis and supporting the decision to include multiple ADL Qis in the array of PAC QIS.

**Fig. 5:**
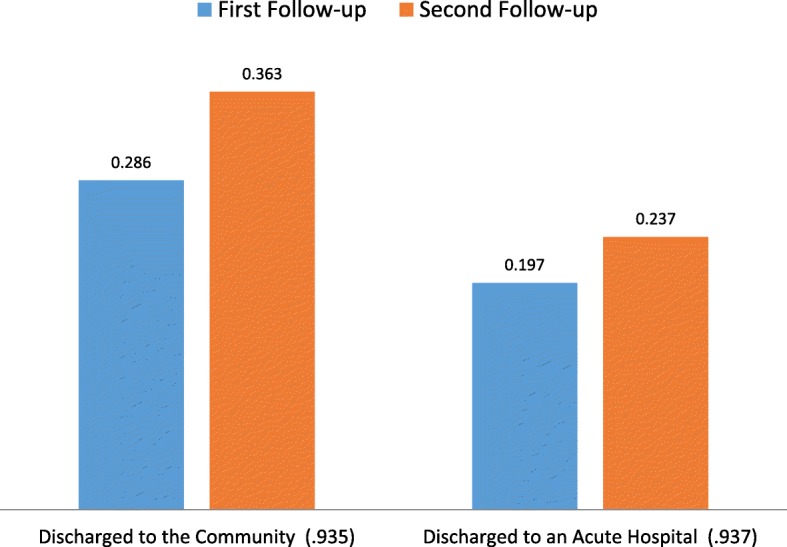
PAC Discharge QIs – Adjusted Rates at 1st and 2nd Follow-ups (Correlation Between 1st and 2nd Follow-up QI)

Figure 5 displays the Discharge Home and Discharge to Hospital PAC QIs. The average discharge home adjusted PAC QI rate for SNFs rose from 29% at the first follow-up to 36% at the second follow-up. The Discharge to an Acute Hospital PAC QI was 20 and 24% by the first and second follow-up, respectively.

#### Summary PAC QI scale

We next created a summary post-acute care quality scale – a scale that pulls together PAC QIs that are related to the discharge to the community PAC QI. All of the functional PAC QIs plus the unsteady gait PAC QI had significant, positive relationships with the discharge QI. The remaining clinical QIs, on the other hand, had low to no correlations with the discharge to the community QI. Thus, a patient’s discharge to the community rests on functional improvement, not on immediate improvements in the clinical areas references by these QIs . .

To create the summary scale all seven of the functional and discharge home QIs were first recoded into a “0” to “2” range and then summed. The final seven item scale had an acceptable internal consistecy, Alpha reliability of 0.835.

The scale range is from “0” (the worst possible score) to “14” (the best possible score). At the first follow-up, the scale had a mean of 6.7 – or about in the mid-point on the scale. At the second follow-up, the means rose 10.50. In terms of using the US facility averages to set quality standards for the summary scale the values are as follows: 20th percentile -- 3; 50th percentile – 7; and 80th percentile – 10.

**Fig. 6:**
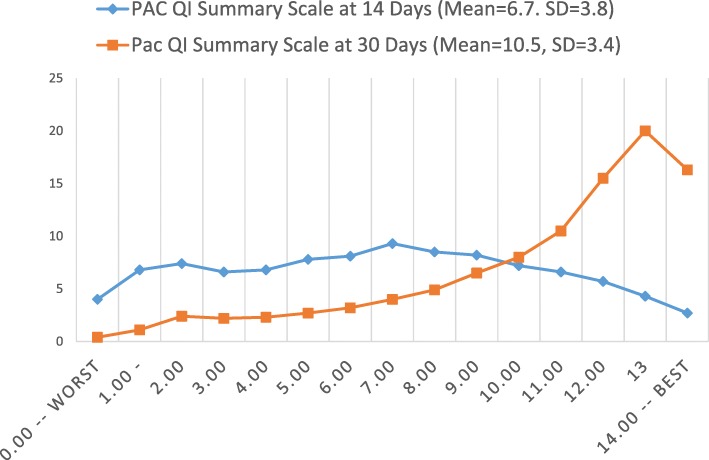
PAC-QI Summary Scale Percent Distribution at 14-Days and 30-Days (Over Time Correlation = .74)

Figure 6 displays the distribution of the PAC QI Summary Scale at 14 and 30 days. Seen from the point of view of the higher scores, the scale brings together sites that do extremely well on all of the ADLs included in the scale. They have a higher rate of discharge of persons to the community. As time progresses the proportion of persons at the high (best) end of the scale increases dramatically – in the top 3 categories the percent rises from 17.7% at the 14-day assessment to 51.8% at the 30-day assessment.

Finally, note that there is considerable interstate variation in the average PAC QI Summary Scale. The 10% of states with the best average performance – including Maine, Minnesotta, Oregon, Rhode Island, and Vermont have an average summary score of 9.63. The 10% of states with the worst average performance -- including Arizona, DC, Kentucky, Louisiana, and Oklahoma have an average score that is about half of this value – or 4.76.

## Discussion

We have presented a broad array of functional and clinical Post-Acute Quality Indicators and demonstrated substantial variability in the measures across an enormous US data set based on person level assessments in 2012 aggregated to the facility level. This dataset has permitted us both to specify stable covariate models and to present proposed standard benchmark values at the 20, 50 and 80% percentage point in the US distribution of PAC facilities. At the same time the applicability of the PAC OIs goes beyond the United States. Other post-acute sites can use these distributions to position their performance against these real-world adjusted standards for each QI. Post-acute care sites are common in many countries, although there are no common standards for guiding quality improvement.

We have shown that PAC sites performance from admission to 14-days is highly related to how they performed from admission to 30 days. Thus, jurisdictions who do not have the 14 day re-assessment, could use admission to 30 days or discharge indicators to judge performance. However, we would strongly encourage assessments at 14 days as an indicator for how well the facility has managed the patient during that early period. Good transitional care from acute hospitals to post-acute care is essential including timely management of associated problems.

PAC functional indicators were brought together to form a coherent summary PAC QI based on their relationship to the discharge to the community PAC QI. The distribution of the PAC QI Summary Scale showed variation across the full scale metric and variation of SNF average scale scores across the US. This translates into significant differences in the availability of superior performing SNFs across the states – with the difference based on differential within state variations in the availability of sites with higher and lower scale scores.

To date there are no international standards or benchmarks for post-acute care within an episode of care but there are a number of smaller international studies [28, 29] Outcomes have been compared from admission to discharge within specific patient groups and re-hospitalization rates have been examined and proposed key indicators [2, 19]. In the US, there is a recent initiative to use a common CARE dataset and common quality indicators across diverse post-acute settings [2]. The measures include re-hospitalization, pressure ulcers and potential functional measures from admission to discharge. None have been specified for early change or change within an episode. Thus the present set of quality indicators remains relevant for public reporting on a proposed interRAI website as well as for internal use of facilities to monitor their own practices.

With increasing use of interRAI assessment tools from which the PAC QIs can be derived (the MDS 3.0, MDS 2.0, LTCF, and interRAI-PAC-Rehab), there is hope that the PAC QIs in this paper have the potential of at least informing the movement toward international standards or benchmarks. We recognize that there will be inter-country difference, just as we saw inter-state differences in the US. With local data, however, one can place a country’s performance within the spectrum of performance as displayed in this paper.

This type of future movement will be facilitated by broader use of interRAI assessment tools across the globe. For example, the MDS 2.0 is in use in a number of countries (e.g., Canada, Finland, and Belgium), the interRAI-LTCF has been adopted in a number of countries (e.g., New Zealand and several Canadian Provinces), and the MDS 3.0 is in use across the US. Thus the PAC QIs described in this paper should be applicable to diverse in-patient post-acute settings including sub-acute and transitional hospital units, geriatric rehabilitation units, convalescent care and free standing in-patient rehabilitation hospitals - anywhere interRAI assessments are used.

Use of common quality measures allow organizations to monitor their performance and compare to similar sites. This study adds to a growing list of quality indicators interRAI based on interRAI assessments for home care, long term care and mental health organizations [30–32]. The post-acute international outcome assessment process is quite varied and one goal of this study is to present a global set of post-acute outcome measures and standards. At the same time we recognize that others will have to address the issue of within country standards for these measures. The great differences across the US states indicate the need for such work.

In this paper we have only begun to test how these PAC QIs may relate to the patients admitted into care. There is much more work to do. For example, previous studies suggested that a higher volume of patients with the same condition (for example, hip fracture) was associated with higher rates of successful discharge home as defined by discharge to the community with no re-hospitalization for 30 days. Investigator found substantial variation in rates of successful discharge to the community varying from 0% at the 25th percentile to 47% at the 75th percentile [33]. Li and colleagues [34] reported higher re-hospitalization rates for SNFs who had lower volumes of post-acute admissions, regardless of diagnosis. Ottenbacher and colleagues [35] found 30-day readmission rates ranged from 5.8 to 18.8% for selected impairment groups across rehabilitation facilities. It would be interesting to examine whether early improvement in functional or clinical domains could account for some of this variation in quality. We ourselves are now looking at identifying patient sub-types who differ in their likelihood of improvement or lack of improvement.

In our view the wide differences in PAC QI Summary Scale scores across states opens the door to inquiries concerning the forces that are driving the better average scores. The nature of a SNF’s post-acute score status depends to some extent on the US state in which the facility is located.

In terms of potential explanations of variations in QIs and related quality one could begin by asking whether therapeutic service intensity plays a role. What is it about the states that lead to such differences in QI rates? How do PAC sites that specialize in certain patient subsets perform vs. sites that take a more average case mix? Are there different cultures of care among the facilities? Do the discharging hospitals set different expectations for the recovery of the patients discharged? Are there differences in the capabilities of the locally available professional personnel? Are there variations in the capabilities of local community service agencies to care for the patient following discharge? Is there something about how local hospitals prepare the patients prior to discharge? Are there differences in the expectations set by local hospitals and physician specialty groups regarding the trajectory of care once the patient is in a SNF? Do local SNFs differ in how they invest the dollars they receive from Medicare under the RUGs model to provide the needed care to these patients?

There are limitations to this study. We did not do multi-level analyses to adjust for size of SNF or geographic location. Both may play a role. We did not ask whether facilities with differing concentrations of blacks, Hispanics, or Native Americans differed in their PAC QI outcome distributions. What we did do was produce truly national PAC QI distributions for one of the largest countries in the world. It is a step, but there is much more to be done.

This paper does present unique information on the general outcome standards that can be expected in PAC settings. For all the PAC quality indicators we presented we have national benchmarking standards at the median, 20th, and 80th percentile (see Additional files 1 and 2). These benchmarks permit SNFs themselves and outside agencies to set targets against what might be possible if the SNF set as its goal to be as good as the best performing sites.

## Conclusions

We have presented a broad array of functional and clinical quality indicators for use in post-acute settings. They are particularly relevant for settings internationally that use interRAI assessments and wish to compare performance of their organization with benchmarks set on this enormous US database. The risk-adjusted indicators target early improvement but may also be used from admission to discharge. They provide an excellent opportunity for furthering our understanding of quality performance in post-acute care.

## Additional files

Additional file 1 ADL and Discharge Improvement QIs – National SNF Distribution at 14 Days. Distribution for all US SNFs of improvement standards at day 14 into the stay (on average): the median point, the 20th percentile (lowest one fifth point for all SNFs), and the 80th percentile (highest or best four fifths point for all SNFs). (DOCX 156 kb)

Additional file 2 PAC Clinical Indicators -- National SNF Distribution at 14 Days. Distribution for all US SNFs of Improvement standards at day 14 into the stay (on average): the median point, the 20th percentile (lowest one fifth point for all SNFs), and the 80th percentile (highest or best four fifths point for all SNFs). (DOCX 153 kb)

## Abbreviations

ADL: Activities of daily living
ALS: Amyotrophic laterals sclerosis
CHF: Congestive heart failure
CMS: Centers for Medicare and Medicaid Services
CP: Cerebral Palsy
CPS: Cognitive performance scale
KR: Kuder-Richardson
LTCF: Long-term care facility
MDS: Minimum data set
MS: Multiple sclerosis
PAC: Post-acute care
QI: Quality indicator
Rehab: Rehabilitation
SNF: Skilled nursing facility
