# Supplementary material for: Developing quality indicators for in-patient post-acute care
Source: BMC Geriatr. 2018 Jul 11;18:161. doi: 10.1186/s12877-018-0842-z (PMC6042453; doi:10.1186/s12877-018-0842-z)
Supplement: Supplementary file 1 [file 12877_2018_842_MOESM1_ESM.docx]

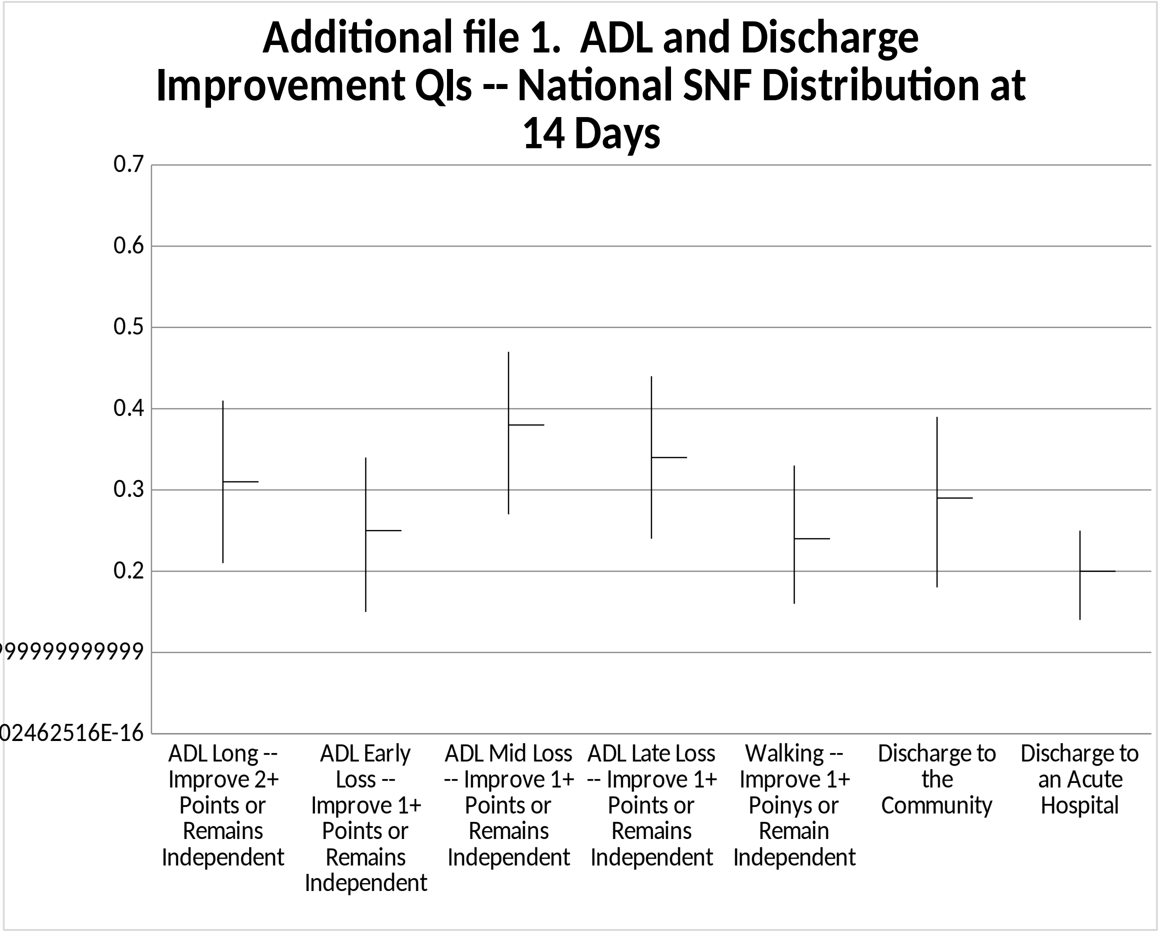
Additional file 1. ADL and Discharge Improvement QIs -- National SNF Distribution at 14 Days
0.7
0.6
0.5
0.4
0.3
0.2
0.0999999999999999
-1.11022302462516E-16
ADL Long -- Improve 2+ Points or Remains Independent
ADL Early Loss -- Improve 1+ Points or Remains Independent
ADL Mid Loss -- Improve 1+ Points or Remains Independent
ADL Late Loss -- Improve 1+ Points or Remains Independent
Walking -- Improve 1+ Poinys or Remain Independent
Discharge to the Community
Discharge to an Acute Hospital
