# Supplementary material for: Developing quality indicators for in-patient post-acute care
Source: BMC Geriatr. 2018 Jul 11;18:161. doi: 10.1186/s12877-018-0842-z (PMC6042453; doi:10.1186/s12877-018-0842-z)

Additional file 2. PAC Clinical and Indicators -- National SNF Distribution at 14 Days

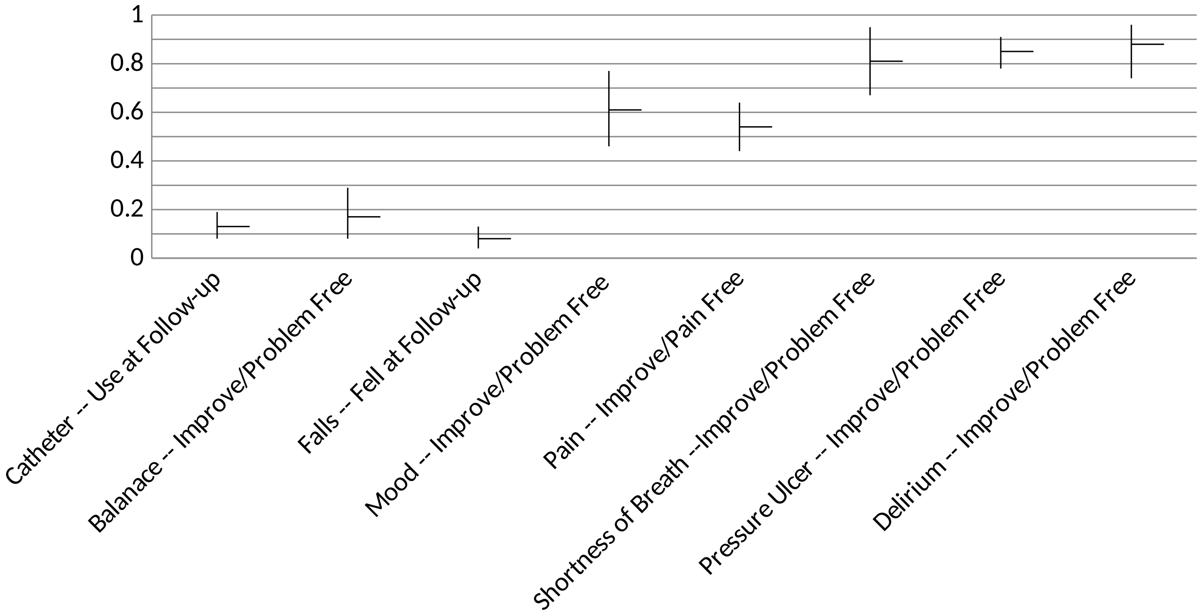

Supplement: Supplementary file 2 [file 12877_2018_842_MOESM2_ESM.docx]
